# Nanomaterials Aiming to Tackle Antibiotic-Resistant Bacteria

**DOI:** 10.3390/pharmaceutics14030582

**Published:** 2022-03-07

**Authors:** Muhammad Usman Munir, Muhammad Masood Ahmad

**Affiliations:** 1 Department of Pharmaceutical Chemistry, College of Pharmacy, Jouf University, Sakaka 72388, Aljouf, Saudi Arabia; 2 Department of Pharmaceutics, College of Pharmacy, Jouf University, Sakaka 72388, Aljouf, Saudi Arabia; mmahmad@ju.edu.sa

**Keywords:** antimicrobial nanomaterial, antibiotic resistance, metallic nanoparticle, bacterial biofilm, multidrug-resistant bacteria

## Abstract

The global health of humans is seriously affected by the dramatic increases in the resistance patterns of antimicrobials against virulent bacteria. From the statements released by the Centers for Disease Control and Prevention about the world entering a post-antibiotic era, and forecasts about human mortality due to bacterial infection being increased compared to cancer, the current body of literature indicates that emerging tools such as nanoparticles can be used against lethal infections caused by bacteria. Furthermore, a different concept of nanomaterial-based methods can cope with the hindrance faced by common antimicrobials, such as resistance to antibiotics. The current review focuses on different approaches to inhibiting bacterial infection using nanoparticles and aiding in the fabrication of antimicrobial nanotherapeutics by emphasizing the functionality of nanomaterial surface design and fabrication for antimicrobial cargo.

## 1. Introduction

The most important current issue related to human health is bacterial resistance against antibiotics. One survey estimates approximately two million cases of severe illnesses caused by bacterial resistance to antibiotics, and 23,000 deaths are reported annually in the U.S. The latest research shows that infection caused by bacteria will be approximately ten million annually in the future [1], which is much greater than recent cancer numbers [2]. Prolonged treatment using antibiotics is required in multidrug-resistant conditions, along with debridement of tissue, but in a small number of cases, the high costs of healthcare and low patient compliance prevent this treatment from succeeding. Estimated reports indicate that in the U.S., societal and health costs annually total almost USD 55 billion [3]. In addition, bacterial cell tolerance is increasing due to the increased use of antibiotics caused by bacterial resistance. It is known from a literature survey from U.S. hospitals that 46–60% of isolated bacterial strains of *S. aureus* are resistant to methicillin and even to vancomycin and carbapenems in some instances [4].

The focal target of antibiotics is mainly on cell wall inhibition, protein synthesis, RNA, and DNA. Bacteria have an intrinsic ability to overcome antibacterial threats by transferring and changing the form of DNA [5,6]. The same microbe can acquire multi-drug resistance that evolves in different organisms. Recent research has shown that super-resistance by resistant bacterial gene NDM-1 in beta-lactam antibiotics causes enzymatic degradation and bacterial resistance against many antibiotics [7]. In current antibiotics, multidrug-resistance in tuberculosis-causing bacteria (mycobacterium) has been reported [3,8]. Resistance against antibiotics will flourish in bacteria, and the growth of more multi-drug-resistant strains of bacteria will be enhanced. It is a formidable situation that requires discoveries of new antibacterial therapies to combat highly resistant bacteria [9].

The promising therapeutic effects of emerging antimicrobial peptides have the potential to reduce the acquisition of bacterial resistance by broad-spectrum activity. The unique characteristics of the amphiphilic topology of the amino acid linkage peptide, namely the poly-cation feature of the head groups, disturb the function of the microbial membrane [10]. Designing antimicrobial therapeutics for building antimicrobial peptides and antibiotics is a potential solution, such as disruption of bacterial cellular machinery, cell membranes, and intracellular components by nanomaterials [7]. The identical features of nanomaterials make them unique candidates and boost the efficacy against multi-drug-resistant bacterial infection. Nanomaterials execute different bactericidal actions, and therefore bacteria face difficulty against therapeutics [11] due to nanomaterial morphology such as size, shape, and surface chemistry. Nanomaterials can penetrate the bacterial membrane with unique drug cargo [12]. Therefore, nanomaterials may be helpful for treatment by improving the therapeutic efficacy through their interaction with the bacterial cell system [13,14].

Research has investigated the efficacy of nanomaterial-based antimicrobials against infection caused by biofilm and planktonic bacteria. A recent study emphasized nanomaterial techniques for multi-drug resistance caused by planktonic bacteria [11,15]. We organized this study into two main focal points: (1) antimicrobial cargo agents, and (2) active therapeutic agents from nanomaterials in antibacterial therapy. Both sections focus mainly on concepts and methodologies for combating multi-drug-resistant bacteria.

## 2. Nanomaterial Interaction with Bacteria

Different factors such as receptor-ligand interactions, Van der Waals forces, electrostatic attraction, and hydrophobicity depend upon bacterial interactions with nanomaterials [7]. The design of novel antimicrobial agents relies on the interaction of bacteria and nanomaterials.

### 2.1. Nanomaterial Penetrating the Bacterial Membrane

**Figure 1:**
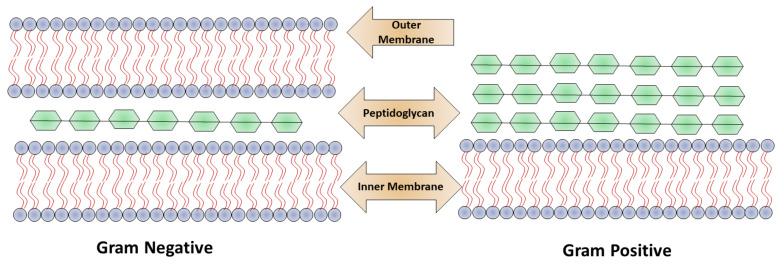
Structural difference between Gram-positive and Gram-negative bacteria.

Based on the bacterial cell wall structure, bacteria are classified mainly into two classes: Gram-positive and Gram-negative as shown in Figure 1. The cell wall structure of Gram-positive bacteria consists of a 15–100 nm thick layer of peptidoglycan and cytoplasmic membrane with techoic acid. In Gram-negative bacteria, the cell wall contains a polymeric chain of techoic acid present in phosphate, which is responsible for the negative charge, and in solution serves as a binding site for divalent cations [5]. Gram-negative bacteria contain a thin (20–50 mm) layer of peptidoglycan, which is followed by a cytoplasmic membrane, and which is protected by lipopolysaccharide consisting of a hydrophobic lipid bilayer. The function of the lipid layer is to reduce the penetration ability of antibacterial agents that are hydrophobic in nature, for example, detergents [12]. Due to the presence of phosphate, the membrane is negatively charged, and in lipopolysaccharides contain carboxylates as a component present on Gram-negative bacteria. The interactions of nanoparticles with the cell walls of the bacteria play a crucial role in the elimination of microbes.

In a study of the interaction of nanoparticles–microbes, Murphy et al. illustrated that CTAB-coated nanospheres and nanorods were equally distributed on Gram-positive *Bacillus cereus*. This type of arrangement created electrostatic interactions between negatively charged techoic acid and positively charged nanomaterials [16]. Alternatively, the substitution of gold particles by mannose bound with the hair-like structure pilin present on Gram-negative *Escherichia coli*. The tiny hair-like structure pilin—a sugar-binding protein rich in lecithin—emanated from the surface of the bacteria bound to the nanoparticles, which were perfectly coated with mannose [17]. Rotello et al. investigated bacterial toxicity by cationic nanoparticles in light of current observations. Similar studies highlighted nanoparticle encapsulation and the membrane structure that the gold nanoparticles formed, which were positive in nature, on the membrane of bacteria [7]. The characteristic feature of AuNP, the core of which was 2 nm in diameter, was that it rapidly lysed Gram-positive bacteria (*Bacillus subtilis*) but was less toxic against Gram-negative bacteria (*E. coli*) [18]. Interactions with bacterial membrane structures and nanoparticles with specific functionalities can result in tubule formation, and membrane defects.

### 2.2. Nanomaterial Mechanism in Providing Antimicrobial Action

Different nanomaterial has different mechanism in combating antibiotic resistance [19,20]. Several NMs have multiple action mechanism to eradicate the microbial resistance, while this resistance can also be inhibited to load an antibiotic in same material [21]. Many antibiotics function via inhibition of the cell wall, disruption of necessary protein, and interfering in DNA replication machinery. Bacteria have the ability to develop resistance against these mechanisms. The primary means of bacterial resistance is to change the antibiotic targets [5,6], such as vancomycin resistance by modification of the components of the bacterial cell wall and tetracycline resistance by alterations in the ribosome structure [7]. For example, enzyme aminoglycoside and b-lactamases are overexpressed by bacteria against different antibiotics. In addition, bacteria can evade multiple antibiotics by the overexpression of efflux pumps. Bacteria, for example, *Chlamydophila pneumonia*, have the ability to reside in the host cells of cellular components from antibiotics and are mainly confined in extracellular space [4,11]. The unique physicochemical characteristics of nanomaterials allow them to cope with antibiotic resistance mechanisms and execute multiple bactericidal novel pathways to attain the activity of antimicrobials. The mode of action of nanomaterials is through leakage of cytoplasmic components by disruption of bacterial membranes [12].

**Figure 2:**
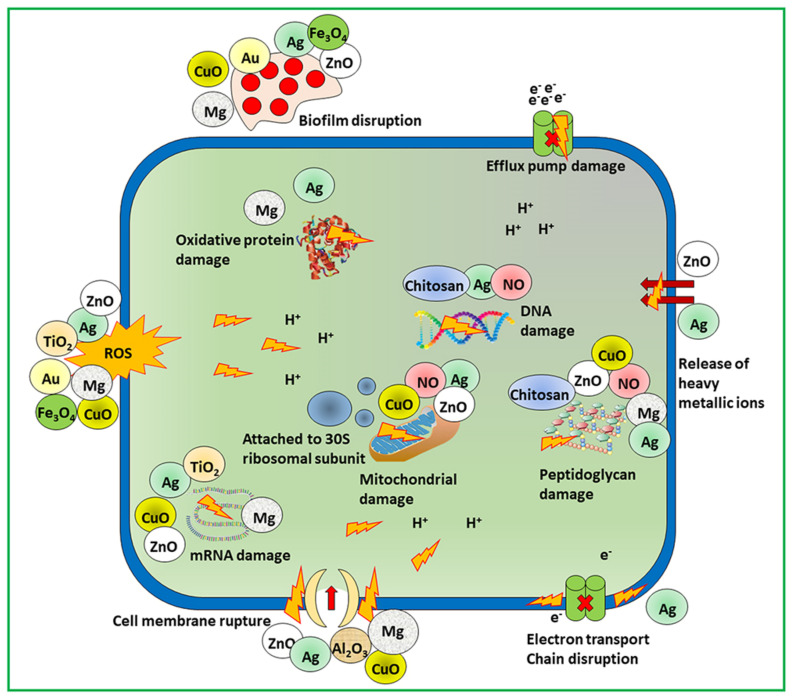
Scheme of mechanistic action of antimicrobial materials to combat microbial resistance. Adapted from [6], Dove Medical Press, 2020.

By puncturing membranes of bacterial cells, nanomaterials become attached to intracellular components, such as enzymes, ribosomes, and genetic material DNA, disturbing the normal function of cell metabolism. Cell death occurs due to electrolyte imbalance, oxidative stress, and enzyme inhibition due to disruption of cellular machinery [7]. The effectiveness of the nanomaterials depends on the size, shape, and core material followed by the bactericidal pathway. Munir et al. summarized the antimicrobial mechanism of action of different nanomaterials including metallic, nitric oxide releasing, and chitosan derivatives nanomaterials as shown in Figure 2 [6].

For example, silver-based nanomaterials contain free silver ions as active agents. The mode of action of silver ions on bacteria is through the electron transport chain and the puncturing of the cell membrane, followed by disruption of DNA. The reactive oxygen species generated by free copper ions from nanoparticles contain copper and target bacterial DNA and amino acid synthesis [22]. Other metal compounds such as titanium dioxide- and zinc oxide-containing nanomaterials kill bacteria by damaging the membrane and producing reactive oxygen species [11,15]. Numerous kinds of nanomaterial cores with different antimicrobial mechanisms can combat drug-resistant superbugs. Non-functionalized nanomaterials have narrow spectra of activity against bacteria. In addition, healthy mammalian cells show fewer therapeutic effects, and their widespread use in biomedical applications is controlled. Nanomaterial surface interactions with bacterial cells enhance the broad-spectrum activity and simultaneously decrease their toxicity in mammalian cells.

## 3. Role of Nanomaterials as Pharmaceutical Active

Due to the unique and versatile physicochemical properties of nanoparticles, they can produce novel therapeutic methodologies. The size of the nanomaterials is similar to bacterial and biomolecular cells and have different types of interactions with small molecules of antibiotics [23]. The smaller size and larger surface area of nanomaterials allow for bacteria–cell multivalent interactions for high loading cargo. Nevertheless, the efflux pumps and overexpression of bacterial drug resistance action can be overcome by the appropriate engineering of nanomaterials [24].

### 3.1. Small Molecule Functionalized Nanomaterials

**Figure 3:**
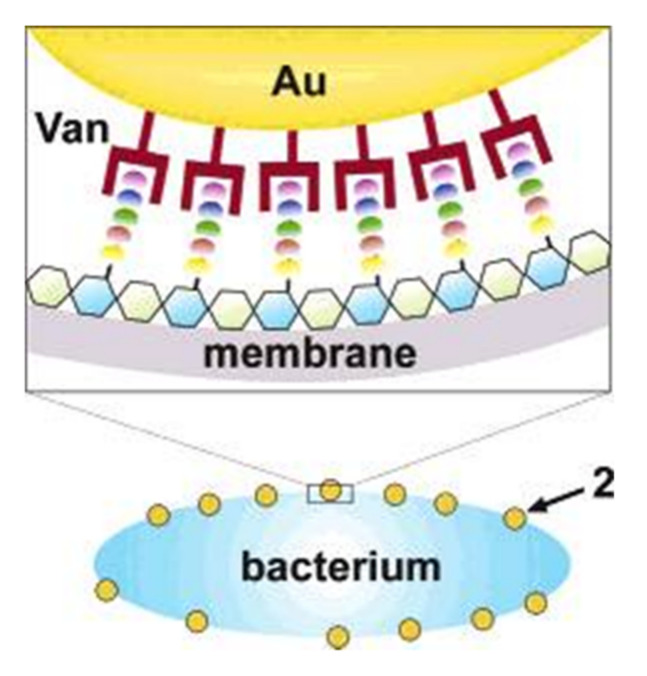
Illustration of a possible multivalent interaction between a Van-capped Au nanoparticle (2) and a VanA genotype VRE strain (hexagons: glycosides; ellipses represent the amino acid residues of the glycanpeptidyl precursor with different colors: *L-Ala* (yellow), *D-Glu* (orange), *L-Lys* (green), *D-Ala* (blue), and *D-Lac* (purple)). Adapted with permission from [25], American Chemical Society, 2003.

Nanomaterials are powerful tools that target the cell membrane of the bacteria and penetrate it. Xu et al. designed a nanomaterial-based antimicrobial through pioneering research in which they fabricated functionalized gold nanoparticles on vancomycin (AuNPs); the purpose of this research was to overcome vancomycin-resistant entero-cocci [25]. In this research, antibiotic vancomycin cystamide through Au–S bonds was conjugated with 5 nm gold nanoparticles, resulting in ∼61 vancomycin molecules per nanoparticles as shown in Figure 3. Vancomycin antimicrobial activity was capped to determine the MIC (minimum inhibitory concentration) for bacterial growth inhibition. The MIC dose of gold capped vancomycin was from 2 to 4 mg/m^−1^. In comparison, vancomycin alone showed a MIC of 64 mg/mL^−1^ against a vancomycin-resistant enterococci bacterial strain. In addition, the functionalized nanomaterials were active against a strain of *E. coli*, but the antibiotic alone was not. In a reported study, an aminoglycoside antibiotic had the ability to absorb onto gold nanomaterials due to interactions between the antibiotic amine group and the gold surface [26].

The antibacterial efficacy of nanoparticles coated with antibiotics was very high against both bacterial species (Gram-positive and Gram-negative). Fayaz et al. formulated a non-covalent conjugation which improved the spectrum activity of silver nanoparticles in the presence of ampicillin antibiotic. A chelating complex was formed between ampicillin (amino and hydroxyl groups) and silver nanoparticles. The actual target of the ampicillin was the bacterial cell membrane, but it was the silver nanoparticle binding to ampicillin, which then bound to the genetic material DNA, that caused bacterial cell death. The antimicrobial activity was enhanced through internalization coupled with the antibiotic inside of the bacterial cell that caused the polyvalent effect on the concentrated antibiotic adhered onto the outer surface of the nanomaterials [27]. The other method is to manufacture antimicrobial nanoparticles, which activate the nanoparticles with smaller molecules that are non-antibiotic in nature. Jiang et al. formulated 3 nm gold nanoparticles coated with amino-substituted pyrimidines that showed antibacterial function against a multiple drug resistance clinical isolator [22]. Positively charged nanoparticles adhered to the surface of the bacterial membrane, which caused membrane rupture and leakage of nucleic acid. Bacterial genetic and protein synthesis inhibition due to binding of the nanoparticles was revealed by proteomic analysis. Nevertheless, the membrane potential and ATPase activities collapsed due to the functionalized nanoparticles, which caused cell death. Rotello et al. controlled the surface activities of nanoparticles combating MDR bacteria using nanoparticle cation functional groups with varying chain length and also non-aromatic and aromatic characteristics.

**Figure 4:**
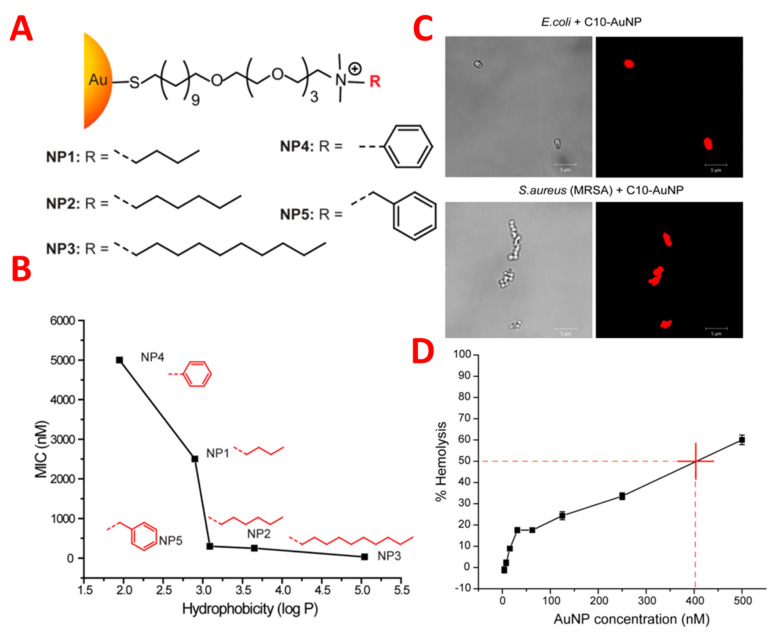
(**A**) Molecular structures of functional ligands on AuNPs. (**B**) MIC values (nM) of AuNPs bearing different hydrophobic surface ligands against laboratory E. coli DH5R. Log P represents the calculated hydrophobic values of the end groups. (**C**) PI staining showing NP 3 (C10-AuNP)-induced bacterial cell membrane damage. Scale bar is 5 μm. (**D**) Hemolytic activity of NP 3 at different concentrations. HC50 was estimated to be ∼400 nM (as denoted by the red cross in figure). Adapted with permission from [28], American Chemical Society, 2014.

The antimicrobial activity of gold-coated nanoparticles was controlled by the structure-activity relationship, which changed the hydrophobicity of nanoparticle surfaces. Nanoparticles are hydrophobic and cationic, and three nanoparticles suppressed the growth of eleven clinical multi-drug-resistant isolates very effectively at concentrations ranging from 8 to 64 nm. These nanoparticles caused the death of the bacterial cell by damaging the bacterial membrane, resulting in leakage of the inner components of the cytoplasmic contents of the cell as shown in Figure 4 [28]. Significantly, these nanoparticles could not produce resistance against the bacteria, even after 20 passages at sub-micro inhibiting concentrations. It is noted that these nanoparticles at 400 nm represent hemolytic activity and have fifty-fold therapeutic selectivity. Considerable delay can be achieved in the resistance of bacteria using nanoparticles in combination with antibiotics in the treatment of planktonic multi-drug-resistant bacteria. The results indicated that hydrophobic nanoparticles with sub. minimum inhibitory concentrations represent a synergistic effect by decreasing antibiotic dosage from 8–16 fold against multi-drug-resistant bacteria [29]. Both Gram-positive bacteria (methicillin-resistant *S. aureus*) and Gram-negative bacteria (*E. coli*, *Pseudomonas aeruginosa*) show efficacy against a combination of nanoparticles and antibiotics. Such a synergetic response acts as a pump inhibitor by the functionalization of nanoparticles and through the bacteria–cell antibiotic complex. The analysis of proteomic bacterial cell outer membrane protein indicates significant efflux protein degradation and the compromising of bacterial cell detoxification. It is noted that nanoparticle use in the mammalian cell was not toxic when used in combinations. The combination of antibiotics with nanoparticles provides a means of combatting multi-drug-resistant bacteria by overcoming certain regulatory issues related to different nano-antimicrobials.

Selectivity in Gram-positive and Gram-negative bacteria can be achieved to determine the surface chemistry of nanoparticles. Grzybowski et al. designed nanoparticles of mixed charges with antimicrobial activity of the Gram-selective type [30]. Nanoparticles were fabricated by different ratios of negatively (MUA) and positively (TMA) charged ligands. The proportions of MUA:TMA in nanoparticle fabrication of 20:80 and 52:48 were used to kill the bacteria selectively at commensurate rates. The study indicated that, on the one hand, cationic ligands help the nanoparticles in their attachment to the surface of bacteria, and on the other hand, anionic ligands interact with cell wall components in which carboxylate head groups compete for hydrogen bonding interactions, which causes lysis of the bacteria by disrupting the structural integrity of the bacterial cell wall. Rotello identified that the charges present on the surface of bacteria are helpful in the determination of nanoparticle antimicrobial activity. Rotello designed nanoparticles using a combination of zwitterions ligands orientation by different charges, one with positive charge present on the outermost layer and another positive charge present inside the ligands’ termini [24]. The antimicrobial activity of nanoparticles, which have outer charges, were more significant compared to the positive charge present inside, with larger or more particles being more effective.

### 3.2. Polymeric Nanomaterials and Polymer-Stabilized Nanomaterials

**Figure 5:**
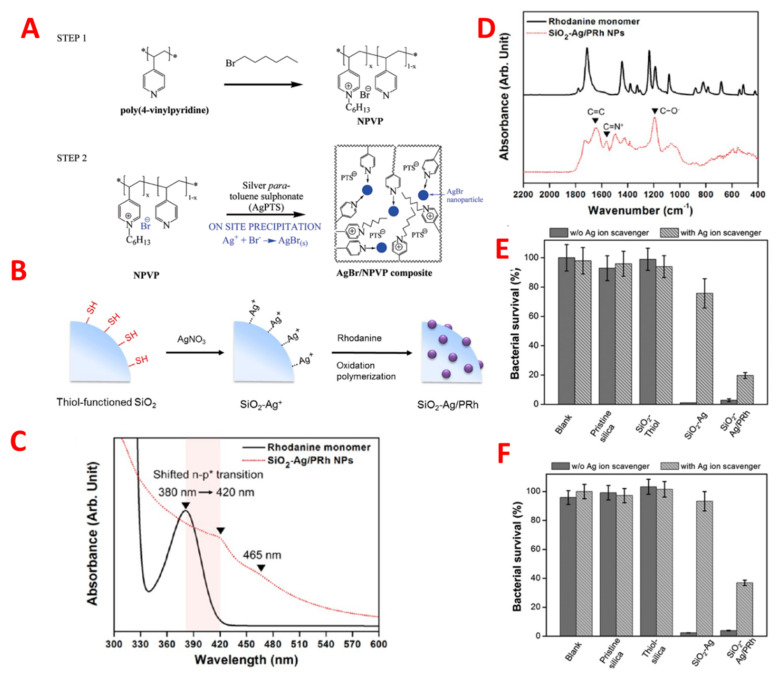
(**A**) Schematic of on-site precipitation method for the synthesis of dual action antibacterial composite material, AgBr/NPVP poly(4-vinylpyridine)-co-poly(4-vinyl-*N*-hexylpyridinium bromide). Adapted with permission from [31], published by American Chemical Society, 2006. (**B**) Schematic Illustration of the Synthetic Procedure for SiO2−Ag/PRh Nanoparticles (**C**) FTIR and (**D**) UV−vis spectra of rhodanine monomer (black solid line) and SiO_2_−Ag/PRh nanoparticles (red dotted line). (**D**,**E**) Antibacterial assessment of four different particles (pristine silica, thiol-silane-treated silica, SiO_2_−Ag, and SiO_2_−Ag/PRh) dispersed in solution and blank water toward *E. coli* and (**F**) S. aureus with or without silver-ion scavenger (100 μL of neutralizer solution) at pH 7.4. Adapted with permission from [33], American Chemical Society, 2013.

Nanomaterials stabilized by polymers have therapeutic effectiveness for an infectious disease against multiple antibiotic resistance. As such, Sambhy et al. indicated that cationic polymers were stabilized by nanoparticles of silver bromide; for example, (AgBr/NPVP), poly (4-vinyl pyridine), and co-poly (4 vinyl *N*-hexylpyridinium bromide) were able to effectively kill both Gram-positive and Gram-negative bacteria. The negative charge caused bacterial membrane lysis by the positively charged cationic polymer as shown in Figure 5A [31]. The release of silver ions controlled the extent of the effect of silver bromide nanomaterial on biocidal activity. In addition, Jang et al. formulated silica polymer-coated nanoparticles (TBAM-co-EGDMA). Polyethylene glycol, a cationic polymer component, enhanced the antimicrobial activity by decreasing the bio adhesion of nanoparticles, which plays a vital role in structural stability and cross-linking of the polymer shell. The effectiveness of the polymer-coated nanomaterial in terms of antimicrobial activity against *E. coli* depends upon the size of the nanomaterial; it was reported that the biocidal effectiveness of 17 nm coated nanomaterial was threefold that of 28 nm particles. Additionally, an increased antimicrobial activity was reported by using a complex coating of silicon, silver, and polyrhodanine on nanoparticles, given that polyrhodanine has strong bactericidal effects [32]. Many species of bacteria were killed by the synergetic effect of antimicrobial nanoparticles, such as silver–silicon with polyrhodanine, by the combination of positively-charged rhodanine as shown in Figure 5B–F [33].

Modifying the magnetic surface of nanoparticles with poly (2-dimethylamino) ethyl methacrylate enhances the therapeutic effect of recyclable antibiotics. *E. coli* is killed 100% by functionalized nanoparticles. The bactericidal activity of nanoparticles increases with increases in the surface area and density and with the stable attachment of antibacterial quaternary ammonium groups on the surface of nanoparticles [34]. The research group of Kyziol found that chitosan is a bactericidal, biocompatible, and biodegradable biopolymer and can be used in the synthesis of gold nanoparticles as a reducing and stabilizing agent. The medium molecular weight (B1280 kDa) chitosan polymer used to stabilize the gold nanoparticles and increase the degree of deacetylation (CS-AuNPs,89T 2%) had a high degree of antibacterial activity, especially against the antibiotic-resistant strains *Pseudomonas aeruginosa* and *Streptococcus aureus*.

The designed CS-AuNPs exhibited selective cytotoxicity by targeting the bacterial membrane. In the eukaryotic cell, they hindered bacterial internalization; with respect to the mammalian tumour and somatic cells, they showed no toxicity [35]. The formulation of nontoxic stabilized silver nanoparticles using a lithium group (AgNPs@PDMAEMA-C4) through the reduction of silver nitrate in the presence of cationic polymer, which was made from (DMAEMA) 2-(dimethylamino)ethyl methacrylate, exhibited strong antimicrobial activity against infection caused by bacteria. A strong synergetic multivalent antibacterial effect was produced by combining AgNPs@PDMAEMA-C4 nanoparticles and polymer. Bacterial cell death occurred due to bacterial cytoplasmic permeability via the membrane and strong inhibition of intracellular enzymatic activity. The in vivo model indicated that the wound infection caused by *Pseudomonas aeruginosa* and *Streptococcus aureus* was healed by nanoparticles [36].

Polymeric materials in aqueous solutions have the ability to formulate organic nanoparticles by themselves and are used to eliminate the bacterial infections [37,38]. By comparison of individual polymer molecules to polymeric nanostructures, it has been noted that the polymer nanostructure formation binding ability to cell membranes was efficient. Yang et al. formulated cationic amphiphilic triblock polysaccharide, which had biodegradable properties that, upon dissolution in water, produced self-assembled nanoparticles that were micellar. The mean zeta potential and diameter of the organic nanoparticles could be optimized by selecting the cationic properties, molecular weight, and hydrophobicity of the poly (carbonate) block. This type of nanoparticle directly targeted the cell wall and membrane and disrupted Gram-positive bacterial species, including methicillin-resistant *Streptococcus aureus*, and stopped bacterial growth. It has been shown from the research that the antimicrobial activity of micellar nanoparticles depended upon the concentration above the critical micellar concentration (CMC) of the polymer. In addition, upon intravenous administration in mice, these micellar nanoparticles did not produce toxicity in the kidneys or liver at specific drug concentrations [39].

Recent research has identified a recyclable novel formulation *N*-halamine derivatized design using the method of cross-linked organic polymethacrylamide molecules, which were synthesized by copolymerization of a methacrylamide surface-free dispersion with cross linker *N*,*N*-methylenebis by using NaOCl for chlorination. This type of nanoparticle, which is a chloramine functionalized nanoparticle, produces reactive oxygen species in organic media, upon association with bacteria, with remarkable specificity in releasing oxidative chlorine [40], especially *N*-halamine nanoparticles specifically coated on the membrane of *Streptococcus aureus*, and the association between nanoparticles and bacteria with highly specific associations.

Rotello et al. recently designed a library of different degrees of hydrophobicity of quaternary ammonium poly (oxanorborneneimides) and also judged the antimicrobial activities [41]. This kind of polymer formed polymeric nanoparticles of 15 nm diameter in aqueous solutions. By increasing the cationic head groups due to alkyl chain hydrophobicity, the polymeric nanoparticles antimicrobial activity could be enhanced. Increasing the internal chain length by 11 carbons led to a 1000-fold decrease of nanoparticles MIC, but when the cationic group hydrophobicity was increased, no change was observed in MICs. Nevertheless, by increasing the cationic group hydrophobicity, the cytotoxicity of mammalian cells was increased. The incredibly effective polymeric nanoparticles (P5) yielded therapeutic activity and decreased hemolysis (HC50/MIC) by as much as 2500, which is five-fold greater than the polymer, as previously reported. These polymeric nanoparticles stop the growth of bacteria of 11 different clinical isolates and act as broad-spectrum antibacterial agents. The bacteria are not able to develop resistance against polymeric nanoparticles for up to 3100 generations, in contrast to conventional antibiotics. Research work indicates that cationic and hydrophobic moieties determine the antimicrobial activity of nanomaterials on polymer structures.

### 3.3. Nanomaterials Functionalization with Biomolecule

The affiliation of biomolecules has been recognized strongly as having complementary specific interactions with bio specificity to nanoparticles. For example, the bonding of peptides on the surface of the gold (CM-S-Au), AMP-conjugated AuNPs showed high loading of B237 peptides on nanoparticles, resulting in bacterial membrane permeabilization. In addition, these nanoparticle peptides eliminated conjugates in colonies of bacteria in systemic infection and chronic wound models [42]. For biological applications, nanoparticles with protein conjugates are used due to their higher stability, increased enzyme loading capacity, less size dispersibility, and efficient design for targeted delivery. In this context, Hahn et al. designed a conjugated lysozyme with zinc oxide nanoparticles (L-ZNPs), which have effective therapeutic activity against bacteria species, especially *E. coli* and *Streptococcus aureus*, by animated ZnO NPs [43].

Other characteristic features of biomolecules are that they have specific binding sites and the ability to be modified genetically by specific attaching groups. The example of biomolecules (protein, antibodies, and DNA) is attributed to nanoparticles for recognition and highly selective characteristic properties, otherwise synthetic materials are impossible to achieve. Siamak et al. showed that DNA stabilized AgNCs by increasing the antibacterial activity, which was tunable by changing the sequence of the oligonucleotide [25,44]. Using fluorescent nanocluster, the sequence tested antimicrobial activity similar to silver nitrate. The researchers formulated an oligonucleotide arrangement containing a trimeric structure (Seq-3) that had a large amount of amino acid cytosine units that showed strong antimicrobial activity against bacterial species growth (Gram-positive and -negative) in the sub-micromolar range. The amino acid cytosine had better binding capability with silver than nucleotide, which provided a more stable and structured biconjugate. Thus, the genetic material was used to stabilize AbNCs, which play a role in antimicrobial activity, while the efficient antimicrobial agent generated the most pre-organized system [45].

Antimicrobial peptides of functionalization of nanoparticles generate new synergistic designs between nanoparticles and stabilizing agents by the combination of multi drug bacterial resistance [10]. Membrane disruption occurs by the antimicrobial peptides (AMPs) that fight against microbial infection. Antimicrobial peptides show impaired clinical translation by the compromised activity of antimicrobials and decrease their stability in proteolytic enzymes and serum. Research by Bi et al. showed that enhancing physical immobilization peptides bound on the surface of nanoparticles increased the longevity and stability of antimicrobial peptides [46]. Bacterial cell wall carbohydrates contain peptidoglycan nanoparticles formulated from b-amylase and substituting succinate or octyl succinate by nisin peptides. These types of nanoparticle peptides have better bactericidal activity against bacteria such as *L. monocytogenes* than free peptide, with duration of activity up to 21 h. Substitution of octyl succinate from nanoparticles increases activity and stability along with strong hydrophobic and electrostatic interactions between peptides and nanoparticles. Another recent approach, by Ferreira et al., was to design the cationic peptide cecropin melittin by covalent bonding in conjugation with gold nanoparticles, which increased the antimicrobial activity with the presence of proteolytic enzyme concentrations at high levels. Additionally, cystine was attached with lysozyme via glutaraldehyde by cross-linking, and in the end, positively charged L-ZNP conjugate was formed. By L-ZNP formation, the antimicrobial activity was enhanced as compared to individual components of lysozyme, ZnO, and nanoparticles. The bacterial membrane disruption occurred by the conjugation of nanoparticles and oxidative stress by ROS caused by Zn^+2^ ion released.

Through lysozyme medicated membranes, membrane pressure is more aggravated and damaging to bacteria cells. In addition, the lysis of the bacteria was achieved by Smeltzer et al. by conjugation of light gold nanoparticles with antibiotics. This process was monitored using a spectrophotometer or photothermal microscope in real-time. The phenomena of bubble formation showed that laser-generated overheating around the cluster of gold nanoparticles was damaging to the bacterial cells. The technology of photothermal microscopy was used especially for lysis of Gram-positive bacteria (*Streptococcus aureus*) by targeting the bacterial cell surface by using different size gold nanoparticles of 10, 20, and 40 nm, which are conjugated with anti-protein antibody [47]. For the development of good nano-therapeutics, the main target in the bacterial cell is the peptidoglycans, and in bacterial species mycobacteria by the trehalose transporter system, in which exogenous trehalose is internalized to the mycobacterium cytoplasm. In bacteria, mycobacterium tuberculosis pathogenicity disrupts the trehalose biosynthesis and plays a key role in trehalose in the bacterial cytoplasm. Kalana et al. stated that nanoparticles conjugated with trehalose especially target *M. smegmatis* over mammalian cells [48].

## 4. Role of Nanomaterials as a Drug Carrier

**Figure 6:**
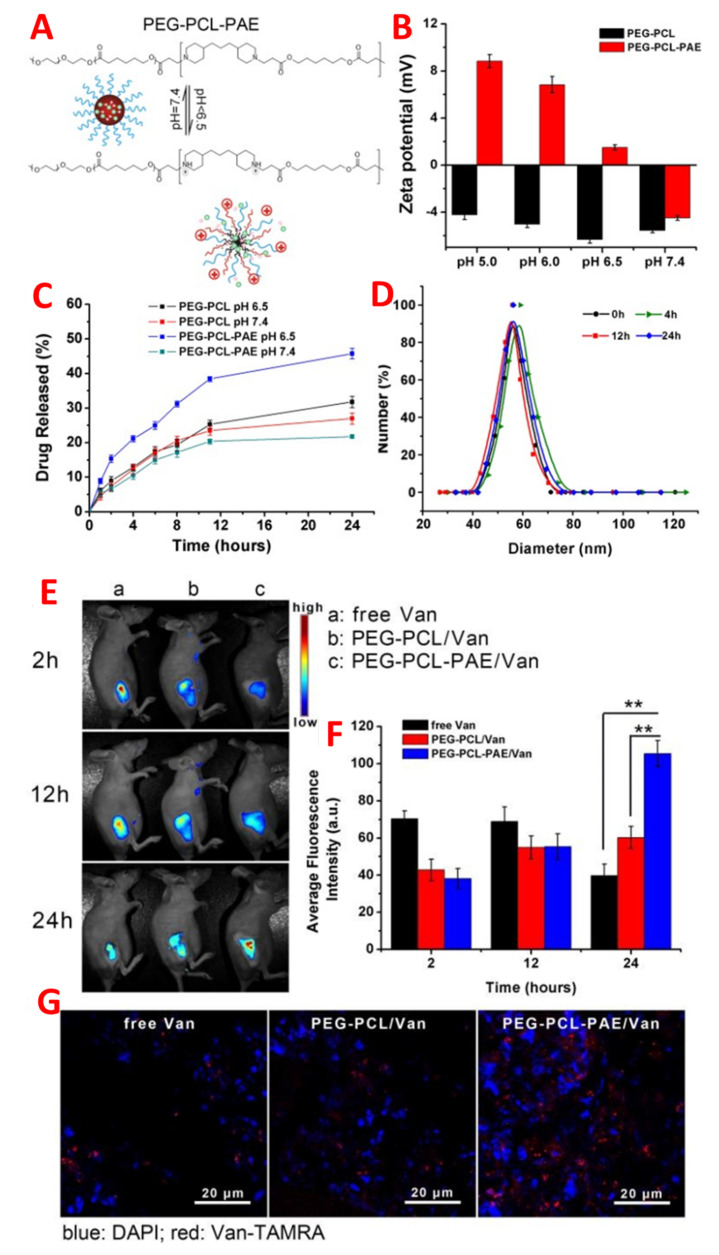
(**A**) Schematic charge conversion of PEG-b-PCL-bPAE/Van nanoparticles due to pH. Zeta potential (**B**) and in vitro Van release (**C**) profiles of PEG-b-PCL/Van and PEG-b-PCLb-PAE/Van nanoparticles under different pH conditions (*n* = 4). (**D**) In vitro stability of PEG-b-PCL-b-PAE/Van nanoparticles in PBS buffer (0.01 M) for 24 h. (**E**) In vivo fluorescence images of inflammation-bearing mice after 2 h, 12 h and 24 h of Van injection and (**F**) statistical analysis and (**G**) representative CLSM images of cutaneous inflammation slices from inflammation-bearing mice after 24 h of Van injection. (*n* = 3, ** *p* < 0.01). Adapted with permission from [51], Royal Society of Chemistry, 2016.

Drug delivery systems, which are based on nanoparticles, can facilitate targeted delivery of the drug at the place of infection, enhance drug retention time in blood, and decrease nonspecific distribution [49,50]. Nanoparticle surface chemistry plays an integral part in nanoparticle solubility in blood and enhances the body’s immune system. Nanovesicles can eliminate the mononuclear phagocytic system from the blood stream unless these delivery vehicles are designed to prevent recognition. Another important process is opsonisation, in which nanoparticle-based delivery of the drug from the biological barrier is conducted. In the mechanism of opsonin, proteins are attached to the blood by nanoparticles, allowing macrophages to eliminate the nanoparticles from circulation through binding to the mononuclear phagocytic system. The therapeutic efficacy of antimicrobials can be enhanced by successful drug delivery vehicle transportation at infection sites [12]. Chu et al. report the charge sensitive nano-system for the persistent and superior in vivo antibiotic delivery as shown in Figure 5 [51]. Figure 6A presents the schematic charge conversion of PEG-b-PCL-bPAE/Van nanoparticles due to pH. Zeta potential, while Figure 6B shows the in vitro Van release. Figure 6C describes the profiles of PEG-b-PCL/Van and PEG-b-PCLb-PAE/Van nanoparticles under different pH conditions (*n* = 4) and Figure 6D shows the in vitro stability of PEG-b-PCL-b-PAE/Van nanoparticles in PBS buffer (0.01 M) for 24 h. In vivo fluorescence images of inflammation-bearing mice after 2 h, 12 h and 24 h of Van injection, and statistical analysis are presented in Figure 6E,F, respectively. Figure 6G indicates the representative CLSM images of cutaneous inflammation slices from inflammation-bearing mice after 24 h of Van injection (*n* = 3, ** *p* < 0.01). Different techniques have been designed to hide nanoparticles from the mononuclear phagocytic system address these limitations. On the other hand, the most popular method is grafting and absorption to a hydrophilic polymer, [52] for example, poloxamers (Pluronic F68) [53], or the coating of nanoparticles with a polysaccharide such as a chitosan. These coating hydrophilic uncharged moieties create a cloud and repel plasma protein and enhance the retention time and circulation time of blood in the circulatory system of the body.

### 4.1. Drug Release Non-Specifically

Antimicrobial agent efficacy is enhanced by using a delivery vehicle targeting antimicrobial and bacterial resistant mechanisms. The disease-producing bacteria evade antibiotics when residing inside the mammalian cell. Due to limited antibiotic availability inside the cell, intracellular infections cannot be treated readily. Another aspect is that the penetration power of nanomaterials inside the cell is higher due to conjugation with high drug loading ability. Biswas et al. utilized the penetrating power of nanomaterials and employed antibiotic tetracycline encapsulated by nanoparticles used in the effective treatment of intracellular *Streptococcus aureus* infections. In designing tetracycline encapsulation, the polymer Chitosan O substituted with a carboxymethyl group was used and positively charged nanoparticles of ∼200 nm in size using the ionic gelation method. The nanoparticles cationic nature led to high uptake inside mammalian cells and binding of tetracycline on the bacterial surface due to the negative charge. From the comparison studies of nano-encapsulated tetracycline and tetracycline alone, it is noted that the survival rate of intracellular Streptococcus decreased from 15% to 2.5% [54].

In the same way, the antibiotic penetration inside the bacteria is less due to lower bacterial outer membrane permeability. In cystic fibrosis patients, up to 90% *Pseudomonas aeruginosa* was isolated due to the high impermeability to aminoglycosides and antibiotics. However, the liposome design from nanovesicles has the ability to very easily transport drugs by fusing with the membrane of the bacteria due to the fluid mosaic model of the bacterial membrane. Omri et al. formulated a nanoliposome vehicle to transport the aminoglycoside to *Pseudomonas aeruginosa*. The liposomal aminoglycoside MIC against *Pseudomonas aeruginosa* was significantly lower than free aminoglycoside and represented good delivery of antibiotic liposomes. The delivery of antibiotics was increased due to bacterial membrane fusion, overcoming the during resistance by microbes and potentially dominating the efflux pumps of bacteria. The bacterial resistance overcoming mechanism, and the stability enhancement of the nanomaterial-based delivery vehicle to cargo in physiological media, such as those chemicals derived from plants, have broad-spectrum antimicrobial activity against MDR strains. However, widespread application is limited due to lower solubility in the aqueous solution [55]. In more recent work, Rotello et al. designed polymers that were bactericidal and stabilized nanosponges with synthetically engineered polymers with cores of essential oil as bases. Polymer (PONI-GMT) acted as an emulsion stabilizer and had a positive charge to enhance the interaction of bacteria. PONI-GMT conjugation between maleimide monomer and disulfide containing dithiol-disulfide made nanosponges degradable at five points. Nanosponges were degradable in the presence of endogenous molecules, such as esterase enzyme and glutathione. Nanosponge (220 nm) activity and stability were maintained even after storage for one year. The nanosponge disrupted the bacterial membrane even at the time of death. In addition, the bacteria were not able to resist the nanosphere for twenty passages, and the MIC of conventional antibiotics were increased more than 1000-fold in some of the passages.

The nanomaterial-based delivery vehicle can regulate the sustained and controlled release of cargo. Friedman et al. formulated a nitrite-loaded saline hydrogel-based composite of nanoparticles to harness nitric oxide antimicrobial activity. These types of composite nanoparticles, namely tetramethoxysilanes (TOMS), were synthesized as sol–gel matrices required to transform sodium nitrite into nitric oxide in the presence of glucose. Other materials, such as polyethylene glycol and chitosan, act as additives to control the sustained release and generation of nitrous oxide upon moisture exposure. These types of nanomaterials exhibited stable and controlled release of nitrous oxide for 24 h and had strong antimicrobial activity against *Streptococcus aureus* bacteria. It was noted that these nanoparticles designed to release nitrous oxide were able to treat methicillin-resistant MRSA in a murine wound model [55]. The co-delivery of multiple antimicrobial nanoparticles can provide a promising platform to exhibit synergetic effects against multiple-drug-resistant bacteria. Gu et al. formulated nanospheres loaded with the antibiotic levofloxacin and silver [56]. They designed mesoporous silver-embedded silica nanoparticles encapsulated by the antibiotic levofloxacin. The silver ions release easily and penetrate into the membrane of the bacteria, simultaneously releasing levofloxacin. Nanoparticles of dual loaded drugs represent synergetic effects against multi-drug-resistant *Escherichia coli*, in both in vivo and in vitro analyses.

### 4.2. Drug Release Facilitated by Stimuli

Effective design of processes for the delivery of nanomaterials requires one to control the mode of release of pharmaceuticals. Such a system of design must respond to stimuli in the microenvironment, react in a patterned way, and mimic the responses of living organisms. Nevertheless, this approach is sophisticated, biocompatible, and complex, such that it can undergo morphological and chemical changes in response to stimuli, such as pH changes and the secretion of enzymes at infection sites.

#### 4.2.1. Drug Delivery Facilitated by pH-Sensitivity

At low pH (4.5), bacterial infections are generally caused by hypoxic conditions. The design of drug delivery systems must be pH-sensitive in the bacterial infection site in acidic environments [57]. Micellar nanospheres are designed using stable polymers by pegylated poly L-histidine, a residue of the histidine protonated at pH 6.5 due to its strong attraction with the negatively charged bacterial cell membrane. Thus, the antibiotic drug vancomycin is encapsulated and, in an acidic nano-environment, is released at the site of infection. This type of nanosphere avoids nonspecific physiological interactions at pH 7.4 by lowering the pH, and the therapeutic activity is increased [58]. In another research study, polyethylene glycol, a charged adaptive base of nanomaterials, poly (b-amino ester), and poly-caprolactone were used to deliver the antibiotic vancomycin for in vivo targeted delivery. The overall cationic charge under acidic conditions via the inherent nanovehicle of negatively charged (PEG-b-PCL,b-PAE) could facilitate the antibiotic vancomycin with increased circulation time in a subcutaneous inflammation model.

#### 4.2.2. Drug Delivery Integrated with Enzyme-Sensitivity

In enzyme-triggered mechanisms, the increase of enzyme expression at the site of infections, including gelatinases and esterase, requires an effective methodology for an antimicrobial drug delivery system. In a recent study of a drug delivery system, entrapment of the antibiotic ampicillin in a nanocarrier polymer was carried out, and at the site of infection was released due to enzymatic degradation (bio-erosion). Following this research, Li et al. reported nanoparticles of supramolecular gelatin (SGNP) as an on-demand antibiotic delivery system via drug release in the presence of gelatinase at the site of a bacterial infection. The red blood cell membrane coated the surface of supramolecular gelatin nanoparticles (SGNPs@RBC), followed by the gelatin nanoparticles being encapsulated by the antibiotic vancomycin (Van SCGNPs@RBC) [52]. The red blood cell membrane coating imparted the properties of biomimetics to (VanCSGNPs@RBC) and significantly enhanced the immune evading capability of the resulting nanocarrier, which could adhere at the site of infection via increased permeability and the effect of retention. At the time of delivery at the site of the infection microenvironment, the membrane of red blood cells on the (VanCSGNPs@RBC) was shown to be a detoxifier that enabled the further intake of the exotoxin that the bacteria produced. At the same time, degradation of the gelatin core by over-expressed gelatin released in the infection microenvironment occurred, and the antibiotic vancomycin was released and was shown to be a very effective therapeutic agent against the pathogenic bacteria [53].

#### 4.2.3. Drug Delivery Triggered by Bacterial Toxin

At the site of infection, due to the abundance of pore-forming bacterial toxins, the target-oriented nanomaterial-based delivery vehicle is used to trigger the toxins from the selective release of the antimicrobial agent. Without harm to the healthy tissues, the drug is launched at the infection site to kill the targeted bacteria using the technology of vancomycin drug liposomes delivered at the site of infection by stabilized phospholipid gold nanoparticles [59]. The liposomes can prevent self-adhesion due to gold nanoparticles decorated with chitosan, with the accompanying release of payload in non-infected (normal) environments. The therapeutic activities of the liposomes are triggered when any toxin-secreting bacteria are near these liposomes and when a toxin is immediately inserted into the liposome. At the site of infection, the effect of the antimicrobial is localized. In general, the role of nanomaterials is to deliver the cargo of antimicrobials in a controlled manner and at a specific site. The high doses of therapeutic drugs have adverse effects, and toxicity issues can be addressed via nanomaterial-based delivery. The distinct advantage of the nanomaterial is that its therapeutic activity makes it unique compared to conventional antibiotics in terms of providing effective treatment and overcoming antibiotic resistance. In short, there is a clinical need for nano-medicine with high safety profiles in terms of both acute and long-term toxicity of these nanomaterials [60].

## Data Availability

Not applicable.
